# The Protective Effect of Ethyl Acetate and *n*-Butanol Fractions of Wine-Steamed Ligustri Lucidi Fructus on Diabetic Nephropathy in Rats

**DOI:** 10.1155/2021/6512242

**Published:** 2021-10-27

**Authors:** Ruqiao Luan, Linlin Sun, Xuelan Zhang, Pan Zhao, Qiao Zhou, Zhihui Zhang

**Affiliations:** ^1^College of Pharmacy, Shandong University of Traditional Chinese Medicine, Jinan 250355, China; ^2^Shandong Provincial Collaborative Innovation Center for Quality Control and Construction of the Whole Industrial Chain of Traditional Chinese Medicine, 4655 Daxue Road, Jinan 250355, Shandong, China

## Abstract

Ligustri Lucidi Fructus (LLF), the dry and ripe fruit of *Ligustrum lucidum* W. T. Aiton (Oleaceae), is a traditional Chinese medicine for nourishing the liver and kidney in clinics for thousands of years. Wine-steamed Ligustri Lucidi Fructus (WLL) can alleviate coolness and smoothness of LLF and enhance the function of nourishing the liver and kidney, so ancient and modern medicine usually used it in clinics. First of all, we prepared the extracts of different polar fractions of WLL to explore the effective fractions and potential mechanisms of WLL in the treatment of diabetic nephropathy (DN). Then, HPLC method was used to determine the contents of 12 active components in WLL and its different polar components. Finally, the potential relationship between 12 active components and physicochemical parameters of DN rats was explored. The pharmacological experiments showed that WLL, ethyl acetate (EtOAc), and *n*-butanol (*n*-BuOH) extracts not only significantly alleviated the clinical symptoms and kidney damage of DN rats but also had obvious anti-inﬂammatory and antioxidant eﬀects. In addition, the results of HPLC analysis showed that the 12 active components of WLL mainly existed in the extracts of EtOAc and *n*-BuOH. The Pearson correlation analysis showed 12 active components and physicochemical parameters had different degrees of correlation. In conclusion, we proved that the extracts of EtOAc and *n*-BuOH were the effective fractions of WLL in treating DN in rats, and they could regulate the levels of inflammatory cytokines and decrease oxidation stress, which provides a basis for further research on the mechanism of WLL in treating DN and provides a pharmacological and chemical foundation for the development of new anti-DN drugs.

## 1. Introduction

Diabetic nephropathy (DN) is one of the systemic microvascular complications of diabetes, which is accompanied by proteinuria [1]. The main pathological changes caused by DN include glomerular hypertrophy, extracellular matrix accumulation, and basement membrane thickening, which lead to glomerular sclerosis or renal interstitial fibrosis, accompanied by proteinuria, renal insufficiency, and dyslipidemia. The pathogenesis of DN is complex, including glucose and lipid metabolism disorders, renal hemodynamics abnormalities, inflammatory response with abnormal cytokine activity, oxidative stress, and other factors [2].

Ligustri Lucidi Fructus (LLF; Figure S1B) is the dried fruit of *Ligustrum lucidum* W. T. Aiton (Figure S1A) in the Oleaceae family [3]. It is mainly found in Hunan, Sichuan, Jiangsu, and Zhejiang provinces in China [4] and is mostly used to nourish the liver and kidney. Nowadays, LLF is often used to treat diabetes and its complications [5]. Studies showed that the extract of LLF can also reduce urinary protein excretion and improve kidney function [6, 7]. The main LLF active components include phenylethanoids, iridoid glycosides, triterpenoids, and others [8]. Phenylethanoid components include salidroside, tyrosinol, hydroxyltyrosinol, acteoside, and echinacoside [9], which have anti-inﬂammatory activities, anticancer activities, lowering blood sugar and blood lipid, and so on [10–12]. The iridoid glycosides components, including specnuezhenide, ligustroside G13 (G13), oleonuezhenide, nuezhenidic acid, neonuezhenide, 1″-O-*β*-D-glucosylformoside, and oleuropein, were reported for lowering blood lipids [13]. Triterpenoids mainly consist of oleanolic acid and ursolic acid, which have antitumor, hepatoprotective, and antiosteoporosis effects [14, 15].

The 2020 edition of Chinese Pharmacopeia recommends that LLF should be immersed in rice wine and then steamed [3]. According to the traditional Chinese medicine theory, wine-steaming LLF can strengthen the function of nourishing liver and kidney. Modern medicine usually uses wine-steamed Ligustri Lucidi Fructus (WLL; Figure S1C) in clinical practice. Studies showed that the contents of specnuezhenide, G13, oleonuezhenide, neonuezhenide, oleuropein, and nuezhenoside decreased after steaming, while salidroside, tyrosol, and hydroxytyrosol contents increased [16, 17]. Pharmacological studies showed that the LLF extract had a good effect on prevention and treatment of DN [18]. However, the effect of WLL on DN has not been reported and the eﬀective fractions of WLL in treating DN in rats are unclear. Therefore, the screening of effective fractions of WLL for treating DN are of great significance to reveal its mechanism of action.

In this study, we researched the effective fractions of WLL for treating DN and preliminarily explored its mechanism of action, which will provide a scientific basis for the clinical application of WLL and lay a foundation for the development of new anti-DN drugs.

## 2. Materials and Methods

### 2.1. Animals

Five-week-old male Sprague Dawley (SD) rats (bodyweight 160 ± 10 g) were purchased from Jinan Pengyue Experimental Animal Breeding Co., Ltd (Shandong, China), and housed under a controlled temperature of 25°C, 12 h light/12 h dark cycle, and relative humidity set at 50 ± 5%. All experiments were approved by the Ethics Review Committee for Animal Experimentation of the Shandong University of Traditional Chinese Medicine (Shandong, China).

### 2.2. Chemicals and Materials

All the raw products were purchased from Shandong Baiweitang Chinese Medicine Pieces Co., Ltd (Jinan, China). 5 samples of LLF were collected from Sichuan and Zhejiang provinces and authenticated by Li Feng, who is a professor at Shandong University of Traditional Chinese Medicine (Jinan, China). Five voucher specimens (Nos. SDCM-YZ2019040501, SDCM-YZ2019040502, SDCM-YZ2019040503, SDCM-YZ2019040504, and SDCMYZ2019040505) were deposited at the Herbarium of Traditional Chinese Medicine, Shandong University of Traditional Chinese Medicine. Methanol, petroleum ether (PE), ethyl acetate (EtOAc), and *n*-butanol (*n*-BuOH) were purchased from Tianjin Fuyu Fine Chemical Co., Ltd. All reagents were of analytical grade. Serum creatinine (Scr), blood urea nitrogen (BUN), 24 h urine protein, high-density lipoprotein cholesterol (HDL-C), low-density lipoprotein cholesterin (LDL-C), triglyceride (TG), total serum cholesterol (TC), superoxide dismutase (SOD), blood nitric oxide (NO), reduced glutathione (GSH), and malondialdehyde (MDA) assay kits were purchased from Nanjing Jiancheng Bioengineering Institute (Nanjing China). ELISA kits for TNF-*α*, IL-10, and IL-1*β* were purchased from Wuhan Genmei Biotechnology Co., Ltd. Streptozocin (STZ) and metformin (Mef) were purchased from Sigma Chemicals Co. (St Louis, MO, USA).

Standard references: hydroxytyrosol, tyrosol, salidroside, acteoside, echinacoside, specnuezhenide, G13, oleonuezhenide, nuezhenidic acid, neonuezhenide, 1″-O-*β*-D-glucosylformoside, and oleuropein analytical standards with purity ≥98% were purchased from Shanghai Yilin Biotechnology Co., Ltd (Shanghai, China).

### 2.3. Preparation of WLL

WLL was processed according to Beijing traditional Chinese medicine decoction piece processing standard [19] in the lab, which means that the LLF was immersed in rice wine within airtight container for 4 h, added to a moderate amount of water, steamed for 24 h until the surface became black, and then air-dried at room temperature.

### 2.4. Preparation of WLL and Its Different Polar Solvent Extracts

WLL was smashed by a pulverizer (DS-Y500 A, Shanghai Dingshuai Electric Appliance Co., Ltd.), accurately weighed 3.0 kg, and then extracted under reflux, respectively, with water, 50% ethyl alcohol, and ethyl alcohol for 2 h each time; the plant/solvent ratio used was 1/8 (w/w). Subsequently, the extracts of 3 times were mixed and concentrated at 60°C under reduced pressure, it was put into a vacuum freeze dryer for freeze-drying, and finally WLL powder was obtained.

The dried WLL powder was accurately weighed and suspended in water and then extracted successively with 3 times volume amounts of PE, EtOAc, and *n*-BuOH until the leachate is colorless and transparent, respectively. Then each solvent extract was concentrated and vacuum-dried at 60°C. Finally, four different polar extracts (PE, EtOAc, *n*-BuOH, and water fraction) were obtained from WLL extract and weighed accurately. The flowchart of the extraction process is shown in Figure S2.

### 2.5. Experimental Procedure

#### 2.5.1. Type 2 DN Induction and Treatment

High-fat-sugar diet (HFSD) combined with STZ regimen was used to induce type 2 DN in rats. A total of 100 rats were acclimatized for 1 week. The normal control group of rats (*n* = 10) were maintained on a conventional standard diet. The rest of the rats (90 rats) were fed on HFSD for 4 weeks. The HFSD consisted of 18% fat, 20% sucrose, 3% cholesterol, and 59% basic food. After that, the HFSD animals fasted for 12 h and then received STZ (50 mg/kg, intraperitoneally) in 0.05 mol/L citrate buffer (pH 4.5). Rats in the normal group received only the same volume of citrate buffer dosed per body mass criteria. Blood glucose levels were determined 72 h after STZ injection and rats with blood glucose levels higher than 16.7 mmol/L were selected for the next experiments [20].

#### 2.5.2. Experimental Design

DN rats were randomly divided into 8 groups (*n* = 10 for each group): normal, model, administered (WLL group, PE extract group, EtOAc extract group, *n*-BuOH extract group, and water extract group; 15 g/kg, calculated by raw drug weight), and Mef group (200 mg/kg). Administered and Mef groups were treated by gastric perfusion once a day for 12 weeks. The normal and model groups were given the same amount of distilled water.

During the treatment period, blood glucose levels of rats were measured every 2 weeks by tail vein blood samples with a portable glucometer after rats had been fasted for 12 h. Urine samples were collected using metabolic cages. 24 h urine samples were collected once every 4 weeks. After the last treatment, food consumption, water intake, and body weight were measured. Then all the animals were transferred to metabolic cages for 24 hours to collect urine and record its volume before being killed. Finally, the rats were sacrificed under anesthesia to obtain kidney and blood samples. The urine samples were separated at 3000 rpm for 5 min and the supernatants were taken and then stored at −80°C until further use. Blood samples were collected from the abdominal aorta and centrifuged at 3000 rpm for 10 min to separate the serum, which was stored at −80°C for further study. Two kidneys were collected and weighed to calculate kidney/body weight index. One kidney was stored in 10% buffered formalin solution for histopathological assessments.

#### 2.5.3. Detection of Biochemical Parameters

The 24 h urine protein was determined by automatic biochemical analyzer, and the contents of Scr, BUN, HDL-C, LDL-C, TG, and TC in serum were determined by automatic biochemical analyzer. Serum level of MDA was assayed in the form of thiobarbituric acid-reacting substances using the kit. The level of serum SOD was investigated using a colorimetric assay kit according to the manufacturer's instructions. GSH in serum was evaluated with an Ellman's reagent-based assay using commercially available kits. NO in the serum samples was determined by Microwell plate method. TNF-*α*, IL-10, and IL-1*β* levels in the serum were detected with Enzyme Linked Immune Sorbent Assay (ELISA) kits according to the instructions of manufacturer.

#### 2.5.4. Kidney Pathological Parameters

A rat kidney was fixed with 10% paraformaldehyde, dehydrated with ethanol, transparented in xylene, and embedded in conventional paraffin and 4 *μ*m thick sections were made. The pathomorphological changes of kidney tissue were observed after staining with hematoxylin and eosin (H&E).

### 2.6. High-Performance Liquid Chromatography (HPLC) Analysis of Active Components in WLL and Its Different Polar Fractions of WLL

The WLL powder and its different polar extracts were dissolved in 50% methanol and filtered through 0.22 *μ*m ﬁlter membrane. Each reference substance was accurately weighed and dissolved in methanol solution to prepare a mixed standard solution. HPLC was conducted using a Kromasil C_18_ column (250 × 4.6 mm id, 5 *µ*m particle size). The mobile phase consisted of acetonitrile (solution A) and 0.1% formic acid in water (solution B), with the following optimized gradient elution: 0 to 10 min, 7% to 12% A; 10 to 35 min, 12% to 25% A; and 35 to 52 min, 25% to 41% A. The flow rate was 1 mL/min, the column temperature was set at 25°C, and the injection volume was 10 *μ*L. The detection wavelength was 240 nm and 280 nm. Our team has established the chromatographic conditions described above.

### 2.7. Statistical Analysis

All data were presented as mean ± standard deviation (SD) and analyzed using one-way analysis of variance (ANOVA). Scheffe's test in the SPSS software 21.0 was used to calculate statistical significance. *P* < 0.05 was considered statistically significant and *P* < 0.01 was considered very significant.

## 3. Results

### 3.1. The DN Model Was Successfully Established

**Figure 1:**
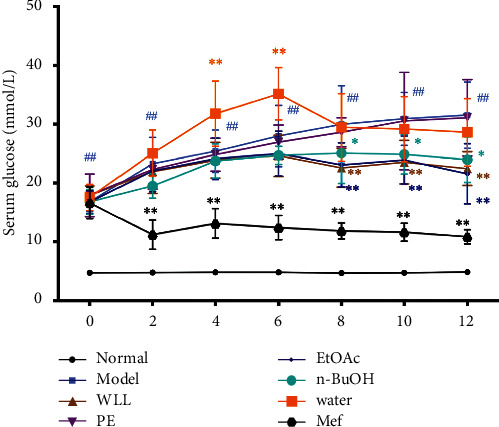
Effects of WLL and its different polar fractions on plasma glucose levels in DN rats. Significant differences with the normal group were designated as ^##^*P* < 0.01; significant differences with the model group were designated as ^*∗*^*P* < 0.05 and ^*∗*^^*∗*^*P* < 0.01.

**Figure 2:**
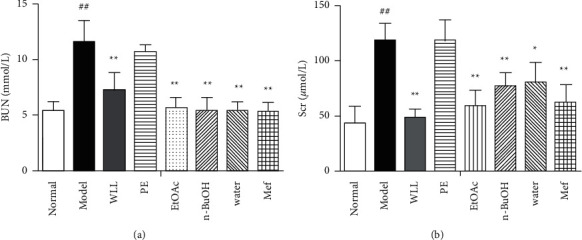
The effects of WLL and its different polar fractions on (a) BUN and (b) Scr in DN rats. Significant differences with the normal group were designated as ^##^*P* < 0.01; significant differences with the model group were designated as ^*∗*^*P* < 0.05 and ^*∗*^^*∗*^*P* < 0.01.

**Figure 3:**
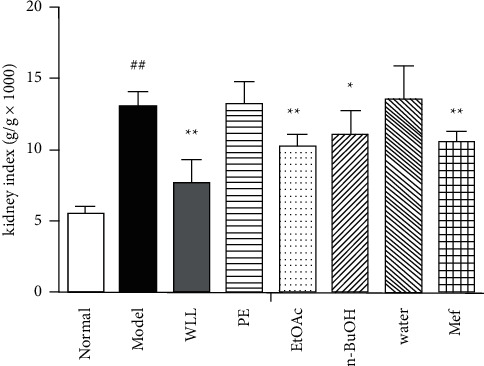
The effects of WLL and its different polar fractions on the kidney index of DN rats. Significant differences with the normal group were designated as ^##^*P* < 0.01. Significant differences with the model group were designated as ^*∗*^*P* < 0.05 and ^*∗*^^*∗*^*P* < 0.01.

Compared with the normal group, after 4 weeks of HFSD and single STZ 50 mg/kg intraperitoneal injection, the rats in the model group showed yellow and matte clothing fur, slowed reaction time, and weight loss, with significantly increased food consumption, urinary output, and water intake (Table 1). After modeling, the blood glucose increased in the model group compared with the normal group (16.80 ± 1.66 vs. 4.76 ± 0.34, mmol/L, *P* < 0.01) as shown in Figure 1. At the 12th week, the 24 h urine protein of the model group was significantly higher than normal group (35.27 ± 3.32 vs. 3.78 ± 0.69, mg/24 h, *P* < 0.01) as shown in Table 2 and the serum BUN, Scr, and kidney index of the model group were significantly increased (*P* < 0.01, Figures 2 and 3) compared with the normal group. Considering the above discussion, the DN model was successful.

### 3.2. Effects on Body Weight, Food Consumption, Water Intake, and Urinary Output in DN Rats

Compared with the normal group, food consumption, water intake, and urinary output increased (*P* < 0.01), whereas body weight decreased in the model group (*P* < 0.01) at the 12th week, which also indicates that the DN model was successful. The body weight of rats in WLL, EtOAc, and *n*-BuOH groups was significantly higher than that in the model group (*P* < 0.01 or *P* < 0.05; Table 1). Compared with the model group, the food intake of rats under treatment with different polar solvent extracts of WLL decreased, in which WLL, EtOAc, and *n*-BuOH extracts promoted significant differences (*P* < 0.01; Table 1). WLL, EtOAc, and *n*-BuOH extracts decreased water intake and urinary output of DN rats compared with the model group (*P* < 0.01; Table 1). Moreover, Mef attenuated changes in body weight, food consumption, water intake, and urinary output in DN rats (*P* < 0.01; Table 1).

### 3.3. Effects on Serum Glucose in DN Rats

In comparison with the normal group, serum glucose of rats increased in the model group (*P* < 0.01), which indicates that the DN model was successful. After 12 weeks, the serum glucose levels of DN rats markedly decreased in WLL, EtOAc, *n*-BuOH, and Mef groups compared with the model group (*P* < 0.01 or *P* < 0.05; Figure 1), while PE and water groups had no notable eﬀect on serum glucose. These results indicate that the WLL, EtOAc, and *n*-BuOH extracts effectively regulated serum glucose levels in DN rats.

### 3.4. Effects on Scr, BUN, and 24 h Urine Protein in DN Rats

The Scr, BUN, and 24 h urine protein were enhanced in DN rats compared with the normal group (*P* < 0.01). In comparison with the model group, the WLL, EtOAc, *n*-BuOH, and water extracts decreased 24 h urine protein, Scr, and BUN (*P* < 0.01 or *P* < 0.05), with a better effect obtained by WLL, EtOAc, and *n*-BuOH extracts. Mef was also effective in regulating Scr, BUN, and 24 h urine protein of rats with DN. However, no alteration in the Scr, BUN, and 24 h urine protein levels occurred in rats that received PE extract. The results are shown in Table 2 and Figure 2.

### 3.5. Effects on Lipid Profiles in DN Rats

**Figure 4:**
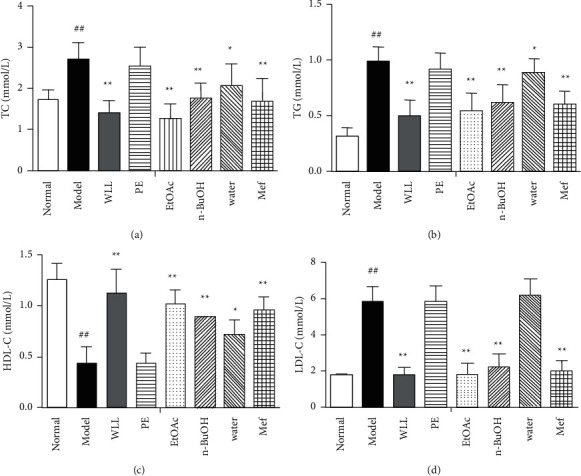
The effects of WLL and its different polar fractions on (a) TC, (b) TG, (c) HDL-C, and (d) LDL-C in DN rats. Significant differences with the normal group were designated as ^##^*P* < 0.01; significant differences with the model group were designated as ^*∗*^*P* < 0.05 and ^*∗*^^*∗*^*P* < 0.01.

In comparison with the normal group, the levels of TC, TG, and LDL-C increased significantly and HDL-C decreased in model group of rats (*P* < 0.01). In the WLL, EtOAc, and *n*-BuOH groups, the levels of TG, TC, and LDL-C were signiﬁcantly lower than those in the model group, while HDL-C increased (*P* < 0.01). The water extracts significantly decreased the levels of TC and TG (*P* < 0.05) and increased HDL-C (*P* < 0.05), but the regulation eﬀect was inferior to that of WLL, EtOAc, and *n*-BuOH groups. The levels of TC, TG, and LDL-C decreased significantly (*P* < 0.01) and HDL-C increased dramatically (*P* < 0.01) in Mef group compared with the model group (Figure 4).

### 3.6. Effects on the Kidney Index in DN Rats

The kidney index (kidney weight/body weight × 1000) increased in the model group compared with the normal group (*P* < 0.01). Compared with the model group, the kidney index in the WLL, EtOAc, *n*-BuOH, and Mef groups significantly decreased (*P* < 0.01 or *P* < 0.05; Figure 4). However, PE extract and water extract had no eﬀect on kidney index.

### 3.7. Effects on Oxidative Stress Parameters in DN Rats

**Figure 5:**
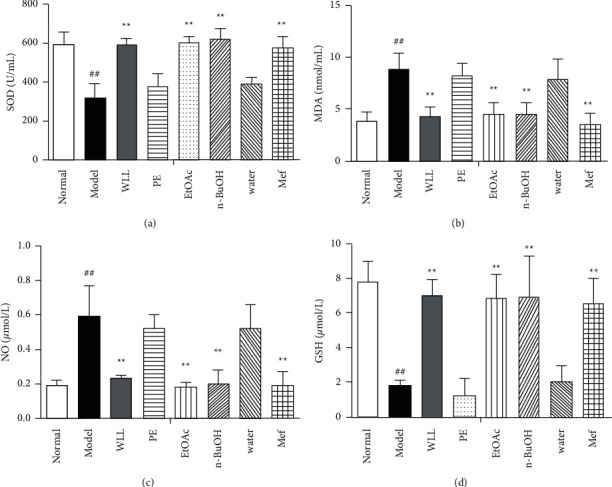
The effects of WLL and its different polar fractions on oxidative biomarkers of DN rats. (a–d) The level of SOD, MDA, NO, and GSH. Significant differences with the normal group were designated as ^##^*P* < 0.01; significant differences with the model group were designated as ^*∗*^^*∗*^*P* < 0.01.

Compared with the normal group, the levels of SOD and GSH activity decreased in the model group (*P* < 0.01), whereas MDA and NO levels increased (*P* < 0.01). Compared with the model group, SOD and GSH activity increased in the WLL, EtOAc, *n*-BuOH, and Mef groups (*P* < 0.01), whereas MDA and NO levels decreased (*P* < 0.01). However, no statistical difference was observed in the PE and water group (*P* > 0.05). The results are shown in Figure 5.

### 3.8. Effects on Inflammatory Cytokines in DN Rats

**Figure 6:**
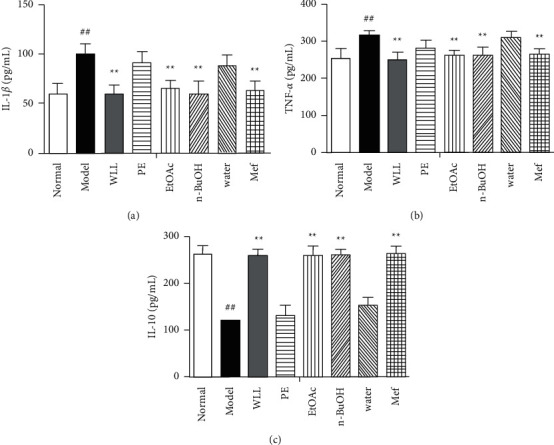
The effects of WLL and its different polar fractions on inflammatory cytokines of DN rats. The serum was collected, and the levels of IL-1*β* (a), TNF-*α* (b), and IL-10 (c) were measured by ELISA. Significant differences with the normal group were designated as ^##^*P* < 0.01. Significant differences with the model group were designated as ^*∗*^*P* < 0.01.

As shown in Figure 6, the concentrations of IL-1*β* and TNF-*α* significantly increased in model group compared with the normal group, while the levels of IL-10 significantly decreased (*P* < 0.01). Compared with model group, EtOAc, *n*-BuOH, and Mef groups significantly restored the levels of the IL-1*β*, TNF-*α*, and IL-10 (*P* < 0.01). In addition, the PE and water extracts had no effect on these cytokines (*P* > 0.05).

### 3.9. Histopathological Findings

**Figure 7:**
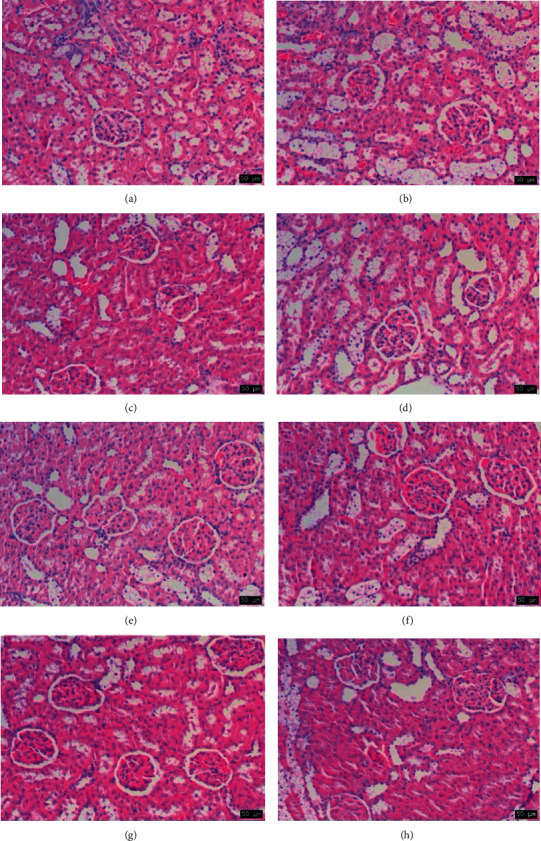
The effects of WLL and its different polar fractions on histopathological findings of DN rats (HE, ×200): (a) normal group, (b) model group, (c) WLL group, (d) PE group, (e) EtOAc group, (f) *n*-BuOH group, (g) water group, and (h) Mef group.

In the normal group, the kidney histoarchitecture under the light microscopic observations showed no abnormal features (Figure 7(a)). The model group showed significant kidney damage, such as glomerular atrophy and localized fibrosis, inflammatory cell infiltration, partial degeneration of the renal tubular epithelium, and interstitial hyperemia (Figure 7(b)), indicating that the DN model was successful. The kidney morphological architecture abnormalities were restored by administering WLL, EtOAc, and *n*-BuOH extracts and Mef for 12 weeks (Figures 7(c), 7(e), 7(f), and 7(h)) compared with model group, indicating that WLL, EtOAc, *n*-BuOH, and Mef groups had significant kidney protective effects. The PE group and water group showed obvious glomerular congestion, renal tubular granule degeneration, and glomerular adhesion, which showed no significant improvement compared with the model group (Figures 7(d) and 7(g)).

### 3.10. HPLC Method Validation

The HPLC method was validated in terms of calibration curve, precision, stability, repetition, and recovery. This method was selective with no obvious interferences. The calibration curves (*n* = 6) showed good linearity; the typical equations of calibration curves and linearity ranges for the twelve analytes containing hydroxytyrosol, salidroside, nuezhenidic acid, tyrosol, echinacoside, neonuezhenide, acteoside, specneuzhenide, 1″-O-*β*-D-glucosylformoside, oleuropein, G13, and oleonuezhenide are shown in Table S1. The results showed that there was excellent correlation between the ratio of peak area and concentration for each component within the linearity ranges. The precision of 12 standard solutions within was lower than 3% RSD. The RSD of the recovery was between 0.41 and 1.55%, the RSD of the repetition test of hydroxytyrosol, salidroside, nuezhenidic acid, tyrosol, echinacoside, neonuezhenide, acteoside, specneuzhenide, 1″-O-*β*-D-glucosylformoside, oleuropein, G13, and oleonuezhenide were 1.28–2.46%, and RSD of stability test was 1.14–2.46%. The twelve analytes were stable during the whole experimental conditions. The results showed that this method can be used in the determinations of WLL and its different polar fractions.

### 3.11. Detection of Active Components in WLL and Its Different Polar Fractions by HPLC

**Figure 8:**
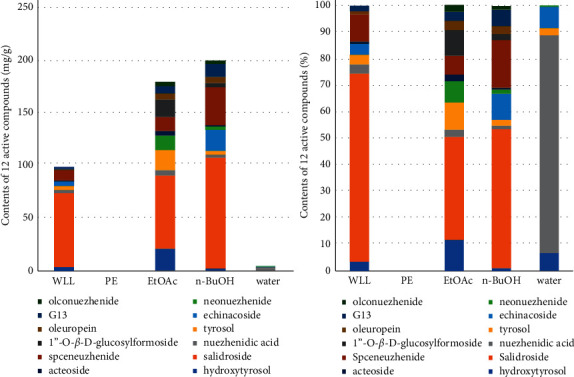
The contents and proportion of 12 active components in WLL and its different polar fractions.

Based on our previous established HPLC method, the distribution and content of 12 active components in different polar fractions of WLL were determined in this study (Figure 8). The study showed that the 12 active components were not detected in PE extract of WLL. Five active components could be detected in the water extract; the content of each compound was very low except nuezhenidic acid. From Table 3 and Figure 8, it could be seen that the 12 active components in WLL mainly existed in EtOAc and *n*-BuOH extracts, indicating that EtOAc and *n*-BuOH had an enriched effect on 12 active components, although the proportion of each compound in WLL and its different polar fractions were quite different (Figure 8).

### 3.12. Correlation Analysis

The potential relationship between 12 active components and physicochemical parameters of DN rat after different polar fractions of WLL treatment was explored by Pearson correlation analysis (Figure S4). As we can see, all the active components were positively associated with HDL-C and body weight and negatively correlated with kidney index, LDL-C, TG, TC, BUN, Scr, 24 h urine protein, blood glucose, food consumption, and water intake. These results indicated that these components had a protective effect on DN in rats. In addition, all active components were negatively associated with MDA (*R* < −0.37), NO (*R* < −0.56) and positively associated with SOD (*R* > 0.53) and GSH (*R* > 0.59), which indicated that these active components in WLL have antioxidant effect. In addition, it is clear that all the active components were negatively associated with TNF-*α* (*R* < −0.56) and IL-1*β* (*R* < −0.51) and positively associated with IL-10 (*R* > 0.63), indicating that these components have anti-inflammatory effect.

## 4. Discussion

DN is a microvascular complication caused by diabetes. In recent years, the incidence rate of DN has been increasing. Oxidative stress and inflammation play an important role in the development of DN [21]. Traditional Chinese medicine has shown better therapeutics in the treatment of DN. As a drug for nourishing liver and kidney, LLF has anti-inﬂammatory eﬀect, reduces blood glucose and blood lipid, has antitumor effect, and regulates the immune system [22]. Previous researches showed that the extract of LLF could prevent and treat DN excellently, but the treatment of DN by WLL has not been studied and its eﬀective fractions are not clear.

Iridoids, phenylethanoids, and triterpenoids are the main components of LLF. Among them, iridoids and phenylethanoids are soluble in water and alcohol, while triterpenoids are soluble in alcohol and insoluble in water. In order to extract all components as much as possible, the water, 50% ethyl alcohol, and ethyl alcohol were used. Then, we used solvent extraction method to prepare extracts of different polar fractions of WLL. The four polar solvents are petroleum ether, ethyl acetate, *n*-butanol, and water in order. The substances extracted from petroleum ether are free triterpenoids and lipid soluble components. The ethyl acetate and *n*-butanol parts mainly contain components with iridoids and phenylethanoids. Most sugars and hydrophilic substances can be extracted by water. This method is simple to operate and can simply separate and enrich the components in the extract of WLL.

STZ intraperitoneal injection combined with high-fat diet induction is a common method to induce a DN model at present. This method is feasible and straightforward, and the model features are similar to early human DN disease manifestations, so it is ideal for studying DN [23–25]. Therefore, we used this method to establish the DN model in rats. The results showed that model rats had the significantly disordered features including increased levels of blood glucose, kidney index, BUN, Scr, and 24 h urine protein, as well as pathological damage, oxidative damage, and inﬂammatory injury, indicating that the DN model was successful.

The progress of DN is related to the increase of overall blood glucose level [26, 27]; the blood glucose levels of rats in model group increased significantly. However, blood glucose levels gradually reduced after 12 weeks of treatment with WLL, EtOAc, and *n*-BuOH extracts and Mef. Therefore, WLL, EtOAc, and *n*-BuOH extracts might prevent the risk of kidney damage by lowering blood glucose. Besides, body weight in model group of rats decreased, and food consumption and water intake increased from their prolonged hyperglycemia. Surprisingly, these symptoms improved with treatment of WLL, EtOAc, and *n*-BuOH extracts and Mef. Furthermore, compared with normal rats, the kidney index of DN rats was increased. In contrast, WLL, EtOAc, and *n*-BuOH extracts and Mef markedly protected DN rats against kidney hypertrophy. A significant alteration associated with the pathological structure of kidney was observed, which revealed the protective effect of WLL, EtOAc, and *n*-BuOH extracts and Mef against kidney damage.

Diabetes can cause glomerular filtration dysfunction, thus resulting in increased urine output and urinary protein content [28]. In addition, BUN and Scr can reflect kidney function. By the end of the experiment, WLL, EtOAc, and *n*-BuOH extracts and Mef reduced the levels of 24 h urine protein, urinary output, Scr, and BUN, which had a significantly protective effect on the kidney of DN. Hyperlipidemia also plays an important role in kidney damage. Kidney disorder is closely linked to high levels of TG, TC, and LDL-C and a low level of HDL-C [29]. When lipid metabolism disorder exceeds the storage capacity of adipose tissue, it will increase LDL-C-mediated oxidative stress and release chemical mediators by glomerular macrophages, leading to the occurrence and development of glomerular sclerosis and the damage of kidney [30]. In this study, we found that WLL, EtOAc, and *n*-BuOH extracts and Mef could reduce the levels of TG, TC, and LDL-C and increase the level of HDL-C, indicating that they had the ability to regulate lipid metabolism. Meanwhile, the extracts of WLL, EtOAc, and *n*-BuOH showed that they had the potential to regulate lipid metabolism similarly to Mef.

Hyperglycemia can trigger inflammation and oxidative stress in vivo, leading to cell death. Effective inhibition of inflammation and oxidative stress in diabetic patients can reduce tissue damage caused by high glucose [31]. SOD and GSH are important antioxidants in the body, which participate in scavenging oxygen free radicals. Free radicals can attack the biofilm and cause lipid peroxidation, resulting in the increase of MDA level. Our results showed that WLL, EtOAc, and *n*-BuOH extracts and Mef inhibited the decrease of SOD and GSH and the increase of MDA and NO, indicating that WLL, EtOAc, and *n*-BuOH extracts and Mef mediated oxidative stress in DN. Inflammatory reaction plays an important role in the occurrence and development of DN [32]. As essential proinflammatory factors, IL-1*β* and TNF-*α* can promote the occurrence of inflammatory reactions and induce renal cell apoptosis through positive feedback [33]. IL-10, an important anti-inflammatory cytokine, usually plays a negative feedback regulation role in inflammatory response [34]. A large number of inflammatory cytokines including TNF-*α* and IL-1*β* were found in the serum of patients with DN [35]. Our research showed that WLL, EtOAc, and *n*-BuOH extracts and Mef could increase the levels of IL-10 and decrease the levels of IL-1*β*, TNF-*α*, and IL-6 in the serum of DN rats, inhibiting the occurrence of inflammatory response.

Among the chemical constituents of LLF, iridoids and phenylethanoids are the components with high content and better pharmacodynamic activity. It has been reported that salidroside has a protective effect on the kidney of DN rats. Salidroside, tyrosol, and hydroxytyrosol have hypoglycemic, anti-inflammatory, and hypolipidemic effects [36–38]. Oleuropein, a component of iridoid glycosides in LLF, also has strong antioxidant effect [39]. Specnuezhenide and salidroside are characteristic components of WLL, which have been used as content determination indexes for quality control of Pharmacopeia 2020 [3]. Therefore, in this study, we carried out quantitative analysis on the 12 components including phenylethanoids and iridoids to explore the material basis of different polar fractions of WLL. The results of HPLC showed that hydroxytyrosol, tyrosol, salidroside, acteoside, echinacoside, specnuezhenide, ligustroside G13, oleonuezhenide, nuezhenidic acid, neonuezhenide, 1″-O-*β*-D-glucosylformoside, and oleuropein are mainly concentrated in EtOAc and *n*-BuOH extracts with high contents. We speculated that these components might be the effective material basis in the treatment of DN. However, we only detected 12 active components in different fractions of WLL. There are still many unknown components in different fractions of WLL that may also contribute to the therapeutic effect, which requires further analysis.

Hydroxytyrosol, tyrosol, salidroside, echinacoside, and acteoside belong to phenylethanoids (Figure S5) which are made of phenylethyl alcohol and caffeic acid. These components have the hydroxyl groups; studies of the phenylethanoid glycosides structure-activity relationship have shown that the hydroxyl groups (especially phenolic hydroxyl groups) in the structure have strong antioxidant activities [40]. In addition, we studied seven kinds of iridoids (Figure S5) that belong to schizocyclic iridoids, all of which contain iridoid alcohols in their parent nucleus. And their basic structures (five-membered ring and one iridoid ring) are essential pharmacophore for anti-inflammatory and hypoglycemic effects [41]. Seven components are esterified at C-11 position and have good anti-inflammatory activity [42]. Hydroxyl group at C-1 position is combined with sugar to synthesize monoglycoside, which enhances the antioxidant effect [43]. Therefore, we hypothesized that these components may play a protective role in DN through the antioxidant and anti-inflammatory pathway, but the mechanism needs to be further studied.

## 5. Conclusion

In this study, the extracts of different polar fractions of WLL were prepared by solvent extraction. Subsequently, we screened the effective fractions of WLL for the treatment of DN in rats through in vivo pharmacodynamic experiments and determined the contents of extracts from different polar fractions of WLL by HPLC. The pharmacodynamic results showed that EtOAc and *n*-BuOH fractions of WLL can signiﬁcantly alleviate the clinical symptoms, reduce pathological injury, and have significant anti-inflammatory and antioxidant effects in DN rats. In addition, the 12 active components in WLL were mainly concentrated in EtOAc and *n*-BuOH extracts. In conclusion, EtOAc and *n*-BuOH extracts have a similar pharmacodynamic substance basis with WLL and are effective fractions of WLL in the treatment of DN. Downregulating the levels of inflammatory cytokines and regulating antioxidant defenses may be part of mechanisms of WLL, but its specific molecular mechanism needs to be further studied.

## Figures and Tables

**Table 1:** The body weight, food consumption, water intake, and urinary output of WLL and its different polar fractions (mean ± SD, *n* = 10).

Groups	Body weight (g)	Food consumption (g/24 h)	Water intake (mL/24 h)	Urinary output (mL/24 h)
Normal	512.83 ± 43.30	26.60 ± 0.53	57.13 ± 1.89	10.35 ± 0.56
Model	225.79 ± 14.16^##^	54.86 ± 2.41^##^	257.63 ± 5.42^##^	118.34 ± 8.35^##^
WLL	280.41 ± 31.58^*∗*^^*∗*^	32.04 ± 1.32^*∗*^^*∗*^	178.27 ± 2.05^*∗*^^*∗*^	17.25 ± 1.69^*∗*^^*∗*^
PE	220.99 ± 31.9	54.05 ± 1.90	257.63 ± 12.96	114.25 ± 7.07
EtOAc	277.66 ± 26.69^*∗*^^*∗*^	34.08 ± 2.78^*∗*^^*∗*^	170.25 ± 11.95^*∗*^^*∗*^	60.78 ± 5.69^*∗*^^*∗*^
*n*-BuOH	264.83 ± 32.36^*∗*^	35.84 ± 2.61^*∗*^^*∗*^	172.88 ± 10.09^*∗*^^*∗*^	82.69 ± 5.86^*∗*^^*∗*^
Water	197.04 ± 40.05	54.68 ± 2.97	246.25 ± 14.08	98.69 ± 8.87^*∗*^
Mef	300.42 ± 27.93^*∗*^^*∗*^	30.48 ± 1.79^*∗*^^*∗*^	169.43 ± 10.65^*∗*^^*∗*^	10.69 ± 1.36^*∗*^^*∗*^

Significant differences with the normal group were designated as ^##^*P* < 0.01; significant differences with the model group were designated as ^*∗*^*P* < 0.05 and ^*∗*^^*∗*^*P* < 0.01.

**Table 2:** The effect of WLL and its different polar fractions on 24 h urine protein of DN rats (mean ± SD, *n* = 10, mg/24 h).

Groups	Week 4	Week 8	Week 12
Normal	5.46 ± 0.39	5.02 ± 0.69	3.78 ± 0.69
Model	25.81 ± 2.84^##^	33.77 ± 5.21^##^	35.27 ± 3.32^##^
WLL	7.52 ± 1.39^*∗*^^*∗*^	8.68 ± 1.21^*∗*^^*∗*^	7.28 ± 0.99^*∗*^^*∗*^
PE	24.04 ± 0.89	31.56 ± 1.75	34.21 ± 3.08
EtOAc	8.98 ± 1.31^*∗*^^*∗*^	11.03 ± 2.17^*∗*^^*∗*^	10.07 ± 0.99^*∗*^^*∗*^
*n*-BuOH	11.14 ± 2.57^*∗*^^*∗*^	12.69 ± 2.39^*∗*^^*∗*^	12.69 ± 1.41^*∗*^^*∗*^
Water	20.23 ± 3.09^*∗*^	27.76 ± 3.46^*∗*^	28.53 ± 1.93^*∗*^
Mef	10.26 ± 1.67^*∗*^^*∗*^	10.89 ± 1.19^*∗*^^*∗*^	10.69 ± 1.53^*∗*^^*∗*^

Significant differences with the normal group were designated as ^##^*P* < 0.01; significant differences with the model group were designated as ^*∗*^^*∗*^*P* < 0.01.

**Table 3:** Contents of 12 active components in WLL and its different polar fractions (mean ± SD, *n* = 5, mg/g).

Components	WLL	PE	EtOAc	*n*-BuOH	Water
Hydroxytyrosol	3.36 ± 0.02	—	20.75 ± 0.02	1.70 ± 0.01	0.25 ± 0.03
Salidroside	69.67 ± 0.25	—	69.67 ± 0.01	105.16 ± 0.02	—
Nuezhenidic acid	3.38 ± 0.04	—	4.97 ± 0.04	2.54 ± 0.03	3.00 ± 0.02
Tyrosol	3.43 ± 0.06	—	18.59 ± 0.09	4.09 ± 0.05	0.10 ± 0.01
Echinacoside	3.88 ± 0.18	—	0.12 ± 0.02	19.75 ± 0.01	0.29 ± 0.02
Neonuezhenide	0.10 ± 0.01	—	14.34 ± 0.13	3.56 ± 0.01	0.02 ± 0.01
Acteoside	0.95 ± 0.02	—	4.36 ± 0.04	1.16 ± 0.01	—
Specneuzhenide	9.93 ± 0.32	—	13.02 ± 0.02	35.92 ± 0.07	—
1″-O-*β*-D-Glucosylformoside	0.12 ± 0.02	—	17.07 ± 0.11	4.36 ± 0.03	—
Oleuropein	0.98 ± 0.03	—	5.88 ± 0.12	5.85 ± 0.04	—
G13	1.92 ± 0.13	—	6.34 ± 0.14	12.13 ± 0.20	—
Oleonuezhenide	0.23 ± 0.01	—	4.16 ± 0.03	3.30 ± 0.04	—

“—“ represents undetected.

## Data Availability

The data used to support the findings of this study are available from the corresponding author upon request.

## Supplementary Materials

Supplementary Materials Figure S1: the *Ligustrum lucidum* W.T. Aiton (A), Ligustri Lucidi Fructus (B), and wine-steamed Ligustri Lucidi Fructus (C). Figure S2: the flowchart of the extraction process. Figure S3: HPLC detection of 12 active components in different polar fractions of WLL. (A) Mixed standard solution in 280 nm; (B) mixed standard solution in 240 nm; (C) extracts of WLL in 280 nm; (D) extracts of WLL in 240 nm; (E) PE extracts of WLL in 280 nm; (F) PE extracts of WLL in 240 nm; (G) EtOAc extracts of WLL in 280 nm; (H) EtOAc extracts of WLL in 240 nm; (I) *n*-BuOH extracts of WLL in 280 nm; (J) *n*-BuOH extracts of WLL in 240 nm; (K) water extracts of WLL in 280 nm; (L) water extracts of WLL in 240 nm. Active compounds: (1) hydroxytyrosol; (2) salidroside; (3) nuezhenidic acid; (4) tyrosol; (5) echinacoside; (6) neonuezhenide; (7) acteoside; (8) specneuzhenide; (9) 1”-O-*β*-D-glucosylformoside; (10) oleuropein; (11) G13; (12) oleonuezhenide. Figure S4: heatmap of correlation between active components and physicochemical parameters in rats after treatment. Figure S5: the chemical structures of 12 components of LLF. Table S1: calibration curve, *r*, and linear range for 12 components (*n* = 6).
