# Supplementary material for: The Protective Effect of Ethyl Acetate and n-Butanol Fractions of Wine-Steamed Ligustri Lucidi Fructus on Diabetic Nephropathy in Rats
Source: Evid Based Complement Alternat Med. 2021 Oct 27;2021:6512242. doi: 10.1155/2021/6512242 (PMC8566045; doi:10.1155/2021/6512242)
Supplement: Supplementary Materials — Figure S1: the Ligustrum lucidum W.T. Aiton (A), Ligustri Lucidi Fructus (B), and wine-steamed Ligustri Lucidi Fructus (C). Figure S2: the flowchart of the extraction process. Figure S3: HPLC detection of 12 active components in different polar fractions of WLL. (A) Mixed standard solution in 280 nm; (B) mixed standard solution in 240 nm; (C) extracts of WLL in 280 nm; (D) extracts of WLL in 240 nm; (E) PE extracts of WLL in 280 nm; (F) PE extracts of WLL in 240 nm; (G) EtOAc extracts of WLL in 280 nm; (H) EtOAc extracts of WLL in 240 nm; (I) n-BuOH extracts of WLL in 280 nm; (J) n-BuOH extracts of WLL in 240 nm; (K) water extracts of WLL in 280 nm; (L) water extracts of WLL in 240 nm. Active compounds: (1) hydroxytyrosol; (2) salidroside; (3) nuezhenidic acid; (4) tyrosol; (5) echinacoside; (6) neonuezhenide; (7) acteoside; (8) specneuzhenide; (9) 1”-O-β-D-glucosylformoside; (10) oleuropein; (11) G13; (12) oleonuezhenide. Figure S4: heatmap of correlation between active components and physicochemical parameters in rats after treatment. Figure S5: the chemical structures of 12 components of LLF. Table S1: calibration curve, r, and linear range for 12 components (n = 6). [file 6512242.f1.docx]

**Supplementary Data**

**The Protective Effect of Ethyl Acetate and n-Butanol Fractions of** **Wine-steamed** **Ligustri Lucidi Fructus on Diabetic Nephropathy in Rats**

Ruqiao Luan,^1^ Linlin Sun,^1^ Xuelan Zhang,^1,2^ Pan Zhao,^1^ Qiao Zhou,^1^ and Zhihui Zhang^1^

*^1^ College of Pharmacy, Shandong University of Traditional Chinese Medicine, Jinan 250355, China*

*^2^ Shandong Provincial Collaborative Innovation Center for Quality Control and Construction of the Whole Industrial Chain of Traditional Chinese Medicine, 4655 Daxue Road, Jinan 250355, Shandong, China*

Correspondence should be addressed to Xuelan Zhang: zhang8832440@sina.com

Ruqiao Luan and Linlin Sun contributed equally to this work and should be considered co-first authors.


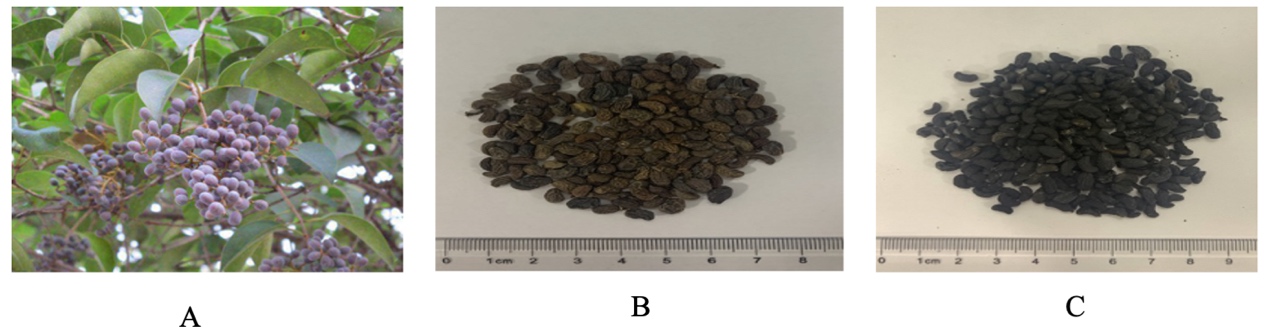


FIGURE S1: The *Ligustrum* lucidum W.T.Aiton (A), Ligustri Lucidi Fructus (B), and Wine-steamed Ligustri Lucidi Fructus(C).


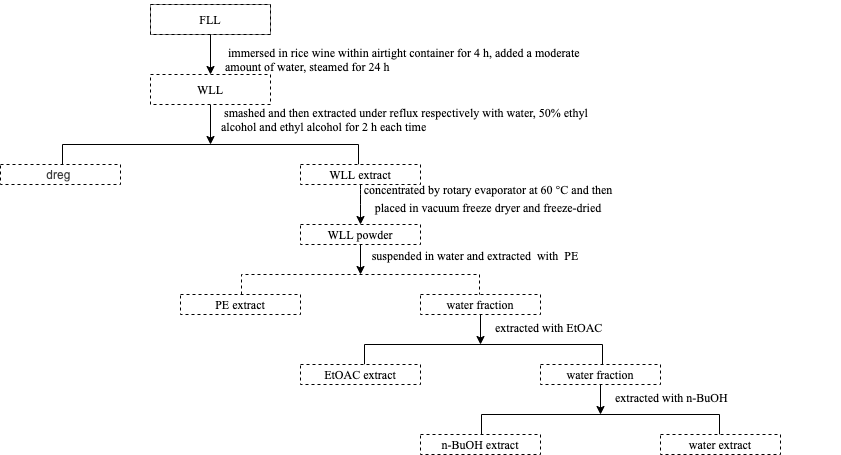


FIGURE S2: The flow chart of the extraction process.


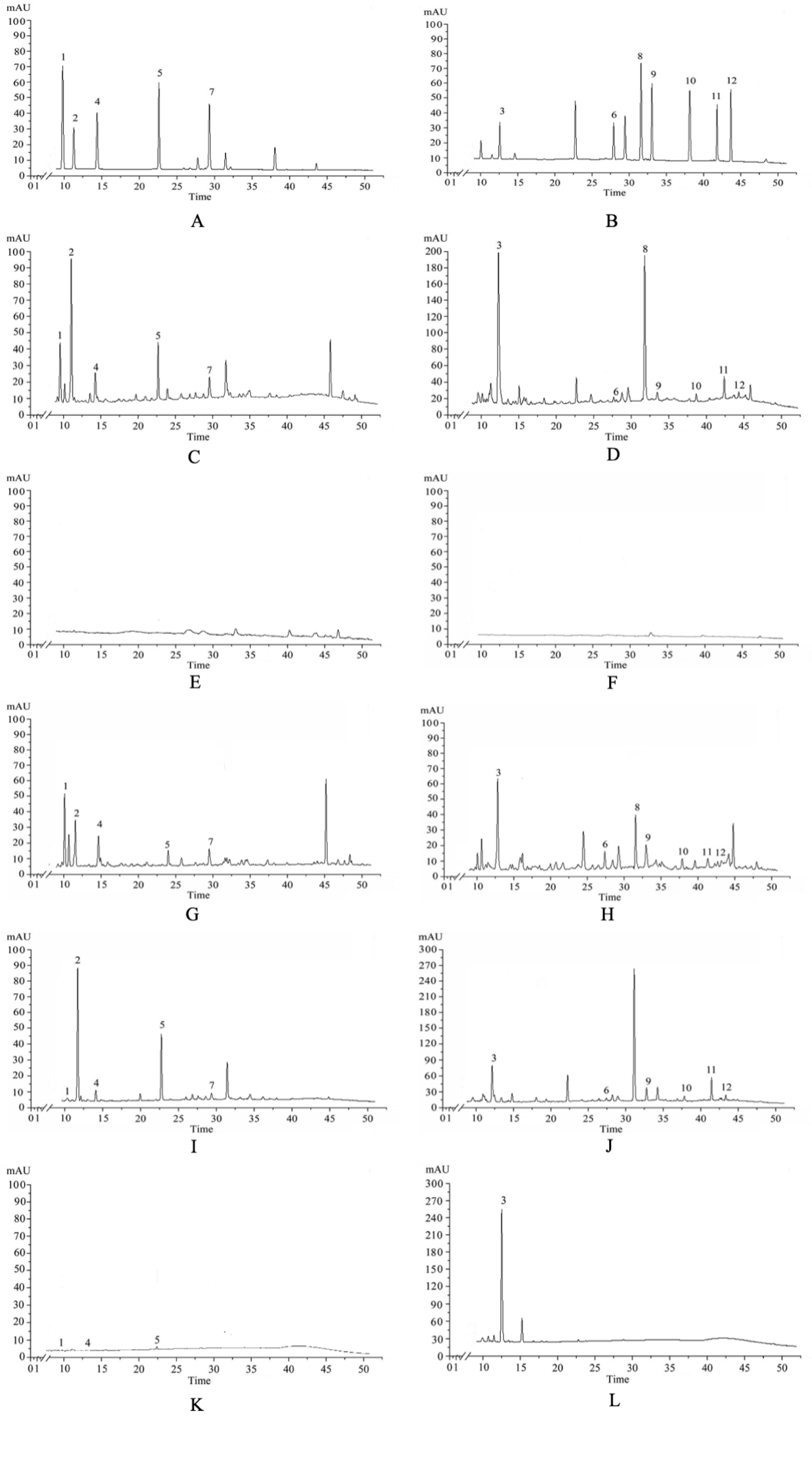


| FIGURE S3: HPLC detection of 12 active components in different polar fractions of WLL. (A) mixed standard solution in 280nm; (B) mixed standard solution in 240nm; (C) Extracts of WLL in 280nm; (D) Extracts of WLL WLL in 240nm; (E) PE extracts of WLL in 280nm; (F) PE extracts of WLL in 240nm; (G) EtOAc extracts of WLL in 280nm; (H) EtOAc extracts of WLL in 240nm; (I) n-BuOH extracts of WLL in 280nm; (J) n-BuOH extracts of WLL in 240 nm; (K) water extracts of WLL in 280nm; (L) water extracts of WLL in 240 nm. Active compounds: (1) hydroxytyrosol; (2) salidroside; (3) nuezhenidic acid; (4) tyrosol; (5) echinacoside; (6) neonuezhenide; (7) acteoside; (8) specneuzhenide; (9) 1''-O-β-D-glucosylformoside; (10) oleuropein; (11) G13; (12) oleonuezhenide. |
| --- |
|  |

*
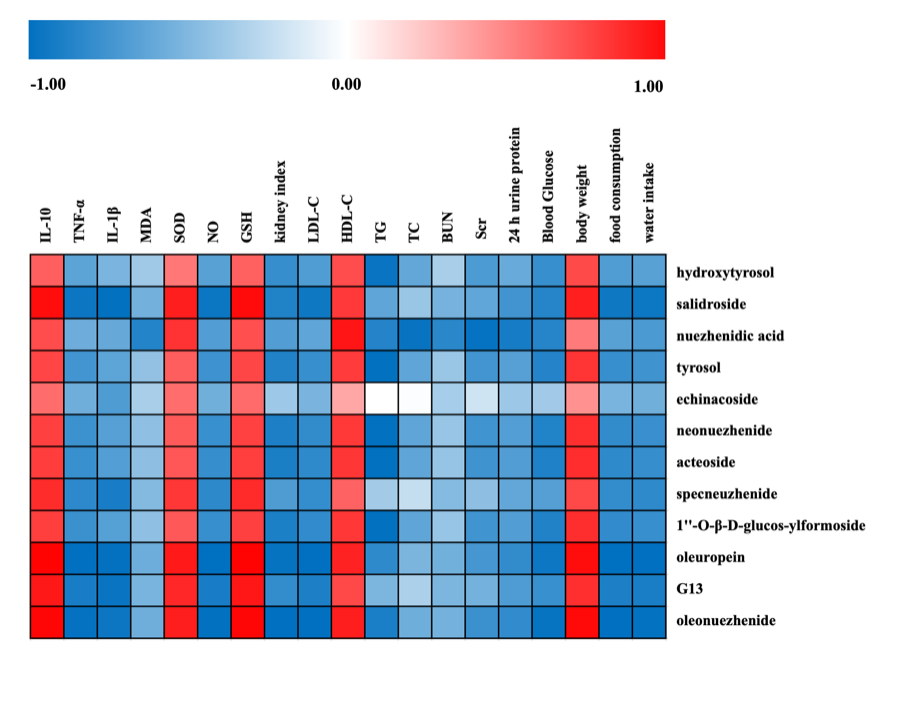
*

| FIGURE S4: Heatmap of correlation between active components and physicochemical parameters rats after treatment. |
| --- |


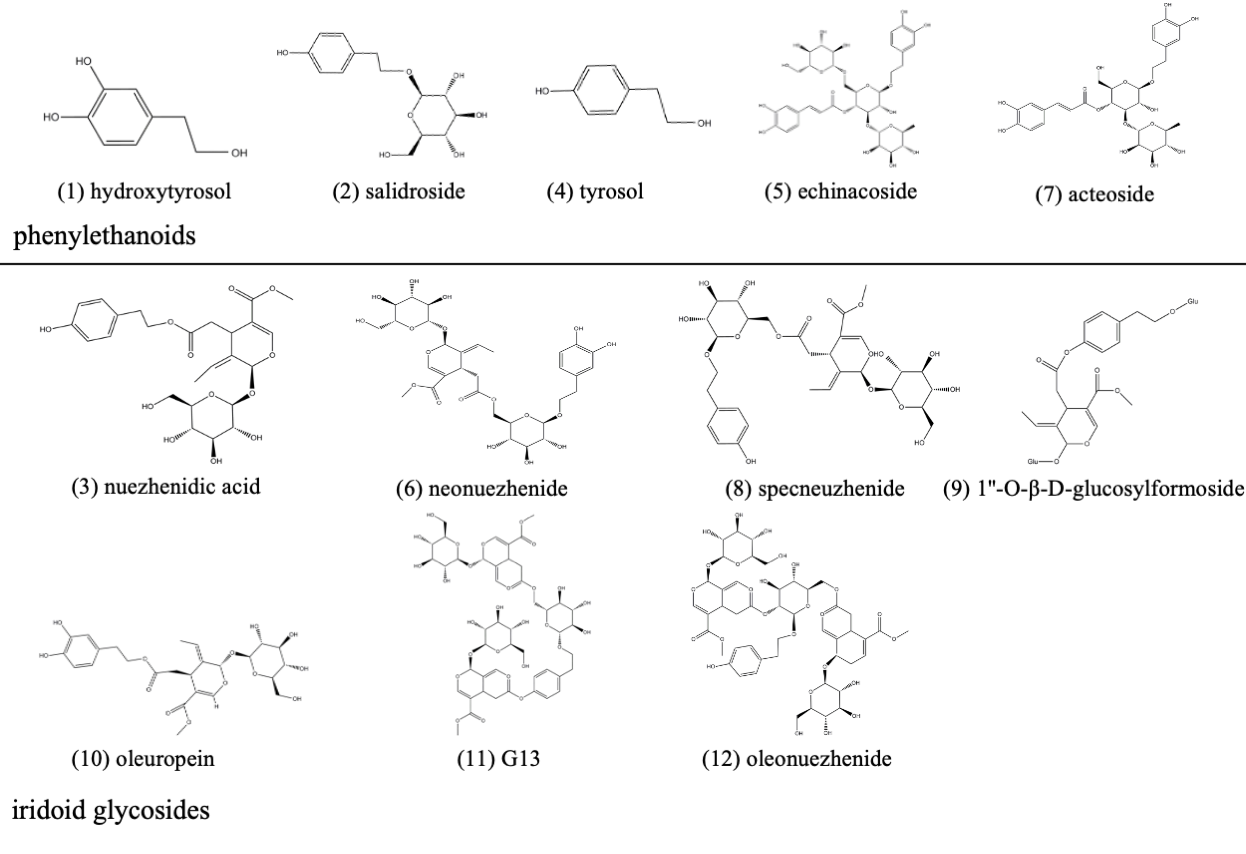


FIGURE S5: The chemical structures of 12 components of LLF.

TABLE S1: Calibration curve, r and linear range for 12 components (n = 6)

| Components | Calibration curves | r | Linear range（µg） |
| --- | --- | --- | --- |
| hydroxytyrosol | Y=383874X+1775 | 0.9997 | 0.004～0.8 |
| salidroside | Y=103513X+786.17 | 0.9998 | 0.009～1.8 |
| nuezhenidic acid | Y= 3000000X+22314 | 0.9997 | 0.01～2.0 |
| tyrosol | Y=228219X+1096.8 | 0.9999 | 0.002～0.4 |
| echinacoside | Y=349687X+123.71 | 0.9999 | 0.004～0.8 |
| neonuezhenide | Y=335408X+9040.2 | 0.9999 | 0.01～2.0 |
| acteoside | Y=647719X-1106.6 | 0.9999 | 0.002～0.4 |
| specneuzhenide | Y=805943X+2933.9 | 0.9999 | 0.003～0.6 |
| 1''-O-β-D-glucosylformoside | Y=735939X+54679 | 0.9999 | 0.005～1.0 |
| oleuropein | Y=489602X+2275.4 | 0.9997 | 0.004～0.8 |
| G13 | Y=489989X+12739 | 0.9991 | 0.002～0.4 |
| oleonuezhenide | Y=742157X+110037 | 0.9995 | 0.002～0.4 |


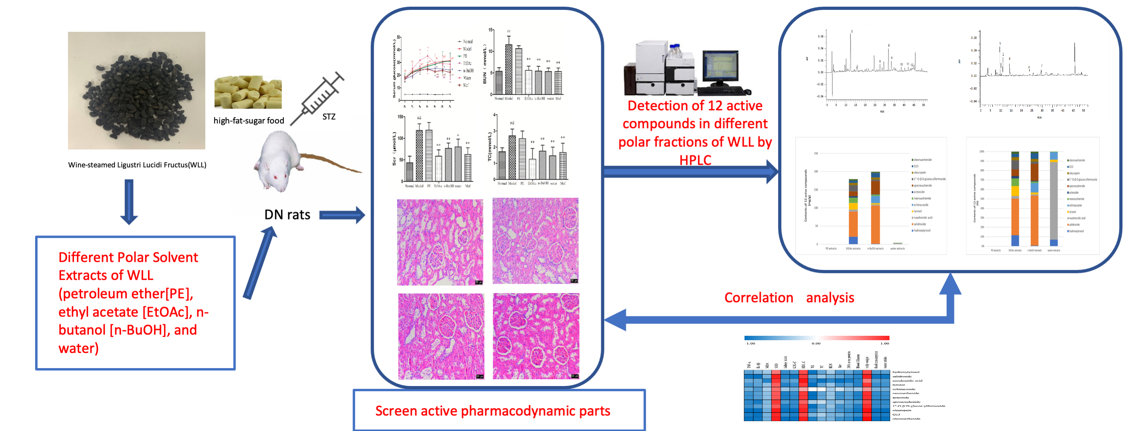


Graphical abstract
